# The human neurosecretome: extracellular vesicles and particles (EVPs) of the brain for intercellular communication, therapy, and liquid-biopsy applications

**DOI:** 10.3389/fmolb.2023.1156821

**Published:** 2023-05-17

**Authors:** Taliah Soleymani, Tzu-Yi Chen, Edgar Gonzalez-Kozlova, Navneet Dogra

**Affiliations:** ^1^ Pathology, Icahn School of Medicine at Mount Sinai, New York, NY, United States; ^2^ Oncological Sciences, Icahn School of Medicine at Mount Sinai, New York, NY, United States; ^3^ Genetics and Genomics, Icahn School of Medicine at Mount Sinai, New York, NY, United States; ^4^ Icahn Genomics Institute, Icahn School of Medicine at Mount Sinai, New York, NY, United States

**Keywords:** extracellular vesicle (EV), exosome (vesicle), brain, therapy, liquid biopsy

## Abstract

Emerging evidence suggests that brain derived extracellular vesicles (EVs) and particles (EPs) can cross blood-brain barrier and mediate communication among neurons, astrocytes, microglial, and other cells of the central nervous system (CNS). Yet, a complete understanding of the molecular landscape and function of circulating EVs & EPs (EVPs) remain a major gap in knowledge. This is mainly due to the lack of technologies to isolate and separate all EVPs of heterogeneous dimensions and low buoyant density. In this review, we aim to provide a comprehensive understanding of the neurosecretome, including the extracellular vesicles that carry the molecular signature of the brain in both its microenvironment and the systemic circulation. We discuss the biogenesis of EVPs, their function, cell-to-cell communication, past and emerging isolation technologies, therapeutics, and liquid-biopsy applications. It is important to highlight that the landscape of EVPs is in a constant state of evolution; hence, we not only discuss the past literature and current landscape of the EVPs, but we also speculate as to how novel EVPs may contribute to the etiology of addiction, depression, psychiatric, neurodegenerative diseases, and aid in the real time monitoring of the “living brain”. Overall, the neurosecretome is a concept we introduce here to embody the compendium of circulating particles of the brain for their function and disease pathogenesis. Finally, for the purpose of inclusion of all extracellular particles, we have used the term EVPs as defined by the International Society of Extracellular Vesicles (ISEV).

## 1 Introduction

**FIGURE 1:**
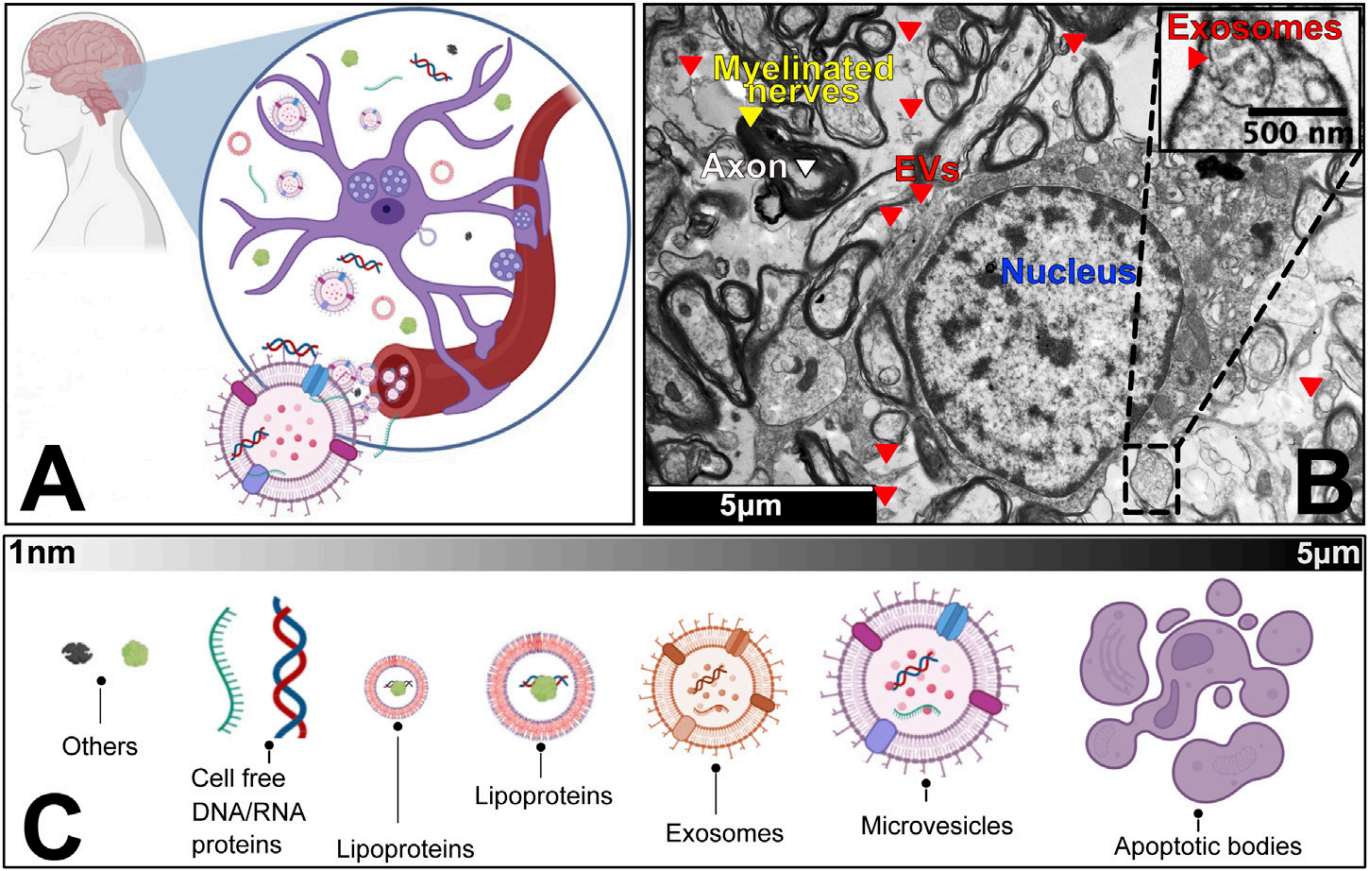
The human neurosecretome. **(A)** Schematic zoom into the universe of extracellular vesicles and particles in the brain microenvironment. We highlight that neurons, microglia and other accompanying cells co-exist with populations of vesicles and particles that are released and up taken by circulatory system. **(B)** Transmission Electron Microscopy of a transversal cut of brain tissue, showing axons (white), myelinated nerves (yellow) and cell nucleus (blue), surrounded by extracellular vesicles (red). Embedded scale shows 5 micro meter distance. **(C)** Overview of the most common subpopulations of EVPs, from smallest (∼1 nano meter) to largest components (5 micro meter).

From eukaryotes to prokaryotes, all cells secrete extracellular vesicles and particles (EVPs) as part of their regular homeostasis, intercellular communication, and cargo disposal (Trams et al., 1981; Pan et al., 1985; Nieuwland and Sturk, 2010). These EVPs may include but are not limited to apoptotic bodies (Kerr et al., 1972), ectosomes/microvesicles (Dalton, 1975; Thery et al., 2018), exosomes (Trams et al., 1981; Pegtel and Gould, 2019), mitochondria-derived vesicles (D'Acunzo et al., 2021; Miller et al., 2022; Popov, 2022; Nakamya et al., 2022), exomeres (Zhang et al., 2018), supermeres (Zhang et al., 2021a), high and low density-lipoproteins (HDL/LDL) (Claude, 1970), ribonucleic proteins (Barrieux et al., 1976), enveloped and non-enveloped viruses (Dalton, 1975; Alem et al., 2021; Dogra et al., 2021), and other cell free proteins/DNA/RNA (Figure 1) (Lo, 2009; Mathivanan et al., 2010; Toden et al., 2020). Recent literature shows that EVPs carry distinct proteo-transcriptomic signatures from their cell of origin. (Chen et al., 2022a). Subsequently, EVPs shuttle around the body as part of a coordinated system of communication between the cells (Simpson and Sonne, 1982; Simpson et al., 2009; Kowal et al., 2014; Thery et al., 2018; Murillo et al., 2019). EVPs are enriched with tissue-specific biomarkers derived from blood, (Dogra et al., 2020; Chen et al., 2022a), urine (Nilsson et al., 2009; Smith et al., 2018), cerebrospinal fluid (Saman et al., 2012; Norman et al., 2021; Sandau et al., 2022), cell culture media (Valadi et al., 2007; Wei et al., 2017; Chen et al., 2022a), and a variety of other fluids (Trams et al., 1981; Valadi et al., 2007; Skog et al., 2008; Nilsson et al., 2009; Simpson et al., 2009; Palanisamy et al., 2010; Bellingham et al., 2012; Raposo and Stoorvogel, 2013; Kowal et al., 2014; DeRita et al., 2017; Shurtleff et al., 2017). These discoveries have brought immense excitement to novel mechanisms of EVP-derived cellular communication, therapy, and liquid-biopsy applications (Fruhbeis et al., 2012; Song et al., 2020; Gaglani et al., 2021).

**FIGURE 2:**
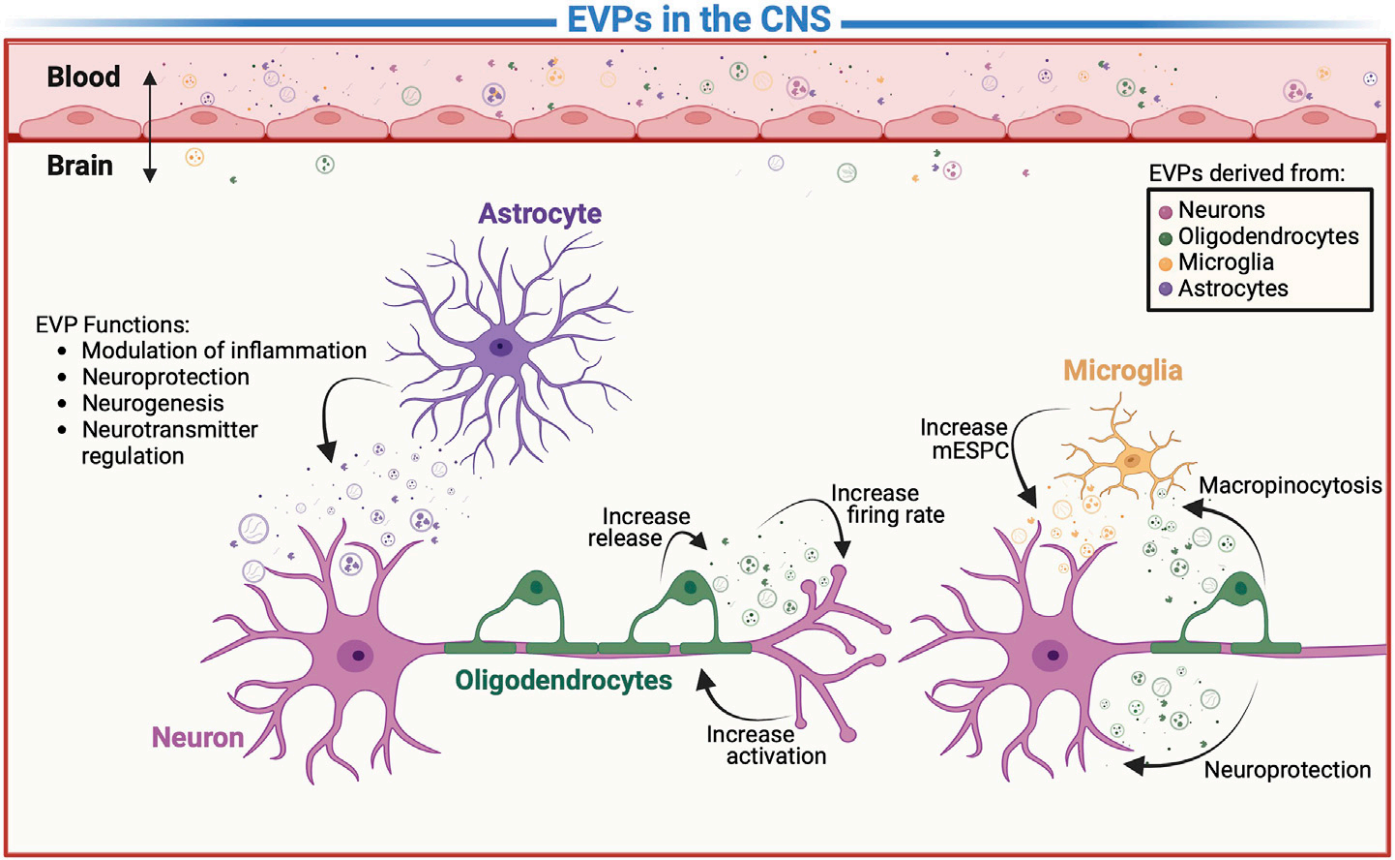
Extracellular vesicles and particles (EVPs) in communication. A detail representation of EVP-mediated mechanism including intracellular communication between the cells of CNS and modulation of molecule transport across the BBB. However, it is unclear which EVPs contribute to etiology of addiction, depression, psychiatric, neurodegenerative diseases, and aid in the real time monitoring of the “living brain”.

Groundbreaking evidence shows that brain-EVPs allow information exchange between the cells of the CNS (Figure 2) (Takeda et al., 2015; Dickens et al., 2017; Song et al., 2020). First described in 2006 (Faure et al., 2006), neurons and astrocytes release EVPs that have a regulatory function at the synapse, which allows intercellular molecular exchange within the brain (Faure et al., 2006; Skog et al., 2008; Song et al., 2020). Recently, plasma, serum and CSF neuronal-enriched EVPs of patients with Alzheimer’s, Parkinson’s, addiction, and glioblastoma were reported to exhibit modulated levels of phosphorylated (p) tau (Kapogiannis et al., 2019a; Palmqvist et al., 2020; Palmqvist et al., 2023), APoE4 (Palmqvist et al., 2023) and Aβ42 (Li et al., 2022; Palmqvist et al., 2023), α-synuclein (Niu et al., 2020), and multiple miRNAs/mRNAs (Lopez et al., 2017; Song et al., 2020) (A detailed list of brain-derived biomarkers is provided on Table 1). Consequently, brain derived EVPs have emerged as key mediators of communication and disposal mechanisms among the CNS. However, there remains a major gap in knowledge with incomplete understanding of the molecular landscape of circulating EVPs that reside in the tissue microenvironment and systemic circulation. To date, most of these secretory particles remain uncharacterized mainly due to lack of technologies to isolate and separate all EVPs, given their variable nanoscale dimensions and low buoyant density (Smith et al., 2018; Murillo et al., 2019; Norman et al., 2021). Thus, the function of several EVPs in disease pathogenesis remains unknown and elusive.

**TABLE 1:** List of brain-derived biomarkers.

Disease	Biomarker	Sample type	Isolation method	References
Alzheimer’s Disease	• Aβ_42_	Plasma Neuron-EVs	2,500 × *g*, (15′at 4°C × 2) + Exoquick® + anti-CD171	Li et al. (2022)
	• P-tau217	Plasma and CSF	Immunoassays	Palmqvist et al. (2020)
	• pSer312	Blood Neuron-EVs	Thromboplastin-D + 3,000 × *g* (20′at 4°C) + ExoQuick® + 1,500 × *g* (20′at 4°C) + L1CAM IP	Kapogiannis et al. (2019a)
	• p-panTyr-IRS-1			
	• p-tau181			
	• p-tau231			
	• Aβ42/Aβ40	Plasma and CSF	Elecsys® plasma prototype immunoassays	Palmqvist et al. (2023)
	• p-tau181			
	• ApoE4			
	• miR-125b-5p	CSF	qPCR	Wiedrick et al. (2019)
	• miR-146a-5p			
	• miR-146b-5p			
	• miR-15b-5p			
	• miR-195–5p			
	• miR-30a-3p			
	• miR-328–3p			
	• miR-23a-3p	Plasma Evs	3,000 × *g* (15′) + Thrombin + 10,000 rpm (5′) + ExoQuick® (System Biosciences) + L1CAM IP	Serpente et al. (2020)
	• miR-223-3p			
	• miR-190a-5p			
	• miR-100-3p			
	• ANXA5	Tissue Neuron-EVs	Sucrose density gradient	Muraoka et al. (2020)
	• VGF			
	• GPM6A			
	• ACTZ			
	• NPTX2	Plasma Neuron-EVs	Thromboplastin-D + 3,000 × *g* (30′at 4°C) + ExoQuick® + L1CAM IP	Goetzl et al. (2018)
	• NRXN2a			
	• AMPA4			
	• NLGN1			
	• SNAP-25	Serum Neuron-EVs	10,000 × *g* (10′at RT) + ExoQuick® + L1CAM IP	Agliardi et al. (2019)
	• N-123 tau, N-224 tau	Serum Neuron-EVs	4,000 × *g* (20′at 4°C) + ExoQuick® + L1CAM IP	Cicognola et al. (2019)
	• BACE1-AS	Plasma EVs	Thrombin + 14,000 × *g* (5′at 4°C) + 3,000 × *g* (15′at 4°C) + ExoQuick®	Wang et al. (2020)
Parkinson’s Disease	• α-synuclein	Plasma Neuron-EVs	2000 × *g* (15′) + ultracentrifugation + L1CAM IP	Niu et al. (2020)
Heroin- dependent patients	• hsa-mia-451a	Plasma Evs	SEC + Exosupur® columns + NTA	Chen et al. (2022b)
Methamphetamine-dependent patients	• hsa-mir-21a	Plasma EVs	SEC + Exosupur® columns + NTA	Chen et al. (2022b)
Major depressive disorder	• pSer312	Plasma Neuron-EVs	Thrombin + 4,500 × *g* (20′at 4°C) + ExoQuick® + L1CAM IP	Nasca et al. (2021)
	• Interferon‐γ	Serum Astro-EVs	3,000 × *g* (30′at 4°C) + ExoQuick®+ ACSA-1	Xie et al. (2022)
	• IL-12p70			
	• IL-1β			
	• IL-2			
	• IL-4			
	• IL-6			
	• TNF-α			
	• IL-17A			
	• PER3	Blood	NanoString + QIAamp RNA blood mini kit	Mamdani et al. (2022)
*Antidepressant response	• MTPAP			
	• SLC25A26			
	• CD19			
	• SOX9			
	• GAR1			
	• miR-144-3p	Blood	NanoString + RT-qPCR	van der Zee et al. (2022)
	• miR-146a-5p	Blood and tissue	mirVana Isolation kit	Lopez et al. (2017)
	• miR-146b-5p			
	• miR-425-3p			
	• miR-24-3p			
Bipolar Disorder	• TNFR1	Plasma Neuron-EVs	Thrombin +4,500 × *g* (20′at 4°C) + ExoQuick® + L1CAM IP	Mansur et al. (2020)
	• NF-κB			
	• Henodeoxycholic Acid	Serum EVs	3,000 × *g* (10′) + qEV column + NSM + NTA	Du et al. (2022)
	• Lysope 18 : 0			
	• Lysope 14 : 0			
	• N-Acetylmethionine			
	• 13-oxoODE			
	• Glycine			
	• 1-Naphthylacetic Acid			
	• 2-Aminoethanesulfonic Acid			
	• D-2-Aminobutyric Acid			
	• Lysopc 18 : 0			
	• Lysopc 20 : 1			
	• Biopterin			
	• Phosphoric Acid			
	• Glucosamine			
	• PAF C-16			
	• miR-484	Plasma EVs	miRCURY Exosome Isolation Kit-Serum/Plasma	Ceylan et al. (2020)
	• miR-652-3p			
	• miR-142-3p			
Schizophrenia	• miR-206	Serum EVs	SEC (qEV iZON Science)	Du et al. (2019)
	• miR619-5p			
	• miR-133a-3p			
	• miR-143-3p			
	• miR-144-5p			
	• miR-499a-5p			
	• miR-3614-5p			
	• miR-941			
	• miR-30c-5p			
	• miR-339-5p			
	• miR-30b-5p			
	• miR-6515-5p			
Human immunodeficiency virus	• S100B	CSF EVs	3,000 × *g* (15′at 4°C) + ExoQuick® + TEM + NTA	Guha et al. (2019b)
Glioblastoma	• miR-182-5p	Serum EVs	SEC (qEV iZONE Science) + TEM +NTA	Ebrahimkhani et al. (2018)
	• miR-328-3p			
	• miR-339-5p			
	• miR-340-5p			
	• miR-485-3p			
	• miR-486-5p			
	• miR-543			
	• IFN-γ	Plasma EVs	3,000 rpm (15′) + OptiPrep™ solution (Sigma-Aldrich) + ultracentrifugation	Cumba Garcia et al. (2019)
	• IL-10			
	• IL-3			
	• B7-1			
	• B7-2			
	• ICOSL			
	• FASN	Plasma EVs	1,000 × *g* (7′) + 100,00× *g* (30′) + ultracentrifugation	Ricklefs et al. (2020)

In this review, we focus on the biogenesis, function, and pathogenesis of the complete EVPs of the neurosecretome. We discuss the extracellular nanoparticles that carry molecular signatures of the brain in its microenvironment and systemic circulation. We elaborate on the current status of technologies used to isolate and separate EVPs of variable sizes and buoyant densities and discuss that every secretory particle may have a distinct cargo and function. It is important to note that the technologies to isolate EVPs are frequently advancing and as a consequence, the complete landscape of EVPs is constantly evolving. Nonetheless, we delve into exploratory and validation studies that have investigated several potential biomarkers of brain diseases circulating within various EVPs. Finally, we employ past literatures and the current landscape of the EVPs to speculate their role in the etiology of addiction (Nakamura et al., 2019; Doncheck et al., 2020; Odegaard et al., 2020; Chand et al., 2021; Odegaard et al., 2022), depression (Saeedi et al., 2021; van der Zee et al., 2022), psychiatric (Du et al., 2022; Nakamya et al., 2022), neurodegenerative diseases (Thompson et al., 2016; Lazo et al., 2021; Noren Hooten et al., 2022; Sandau et al., 2022), and application in real time monitoring of the “living brain”. (Odegaard et al., 2020; Nakamya et al., 2022; van der Zee et al., 2022).

## 2 Extracellular vesicles, particles, and their subtypes

As part of the neurosecretome, we depict an overview of extracellular vesicles, particles, and their diverse subtypes (Figure 1). As a matter of clarity, we begin with the largest vesicles and end with the smallest known particles.

### 2.1 Large vesicles (microvesicles, apoptotic bodies, ectosomes, oncosomes, and beyond)

#### 2.1.1 Microvesicles

Cell activation and cytokine stimulation triggers the formation of microvesicles (MVs) from the plasma membrane that are packaged with cellular components and released into the extracellular environment (Basso and Bonetto, 2016; Greening et al., 2017). The membrane budding formation is initiated by the translocation of phosphatidylserine to the outer membrane (Combes et al., 2010; Basso and Bonetto, 2016). Actin-myosin interactions complete the budding process by contracting cytoskeletal structures (Muralidharan-Chari et al., 2009; Basso and Bonetto, 2016). Consequently, the membrane proteins found on MVs would resemble the receptors and proteins found on specific regions of the plasma membrane of the cell of origin. (Doyle and Wang, 2019). This makes identification of the universal MV markers for all cells types a strenuous challenge (Lotvall et al., 2014; Thery et al., 2018). In addition, the use of markers is extremely essential to identifying MVs since their size range (50–2000 nm) overlaps with that of apoptotic bodies and exosomes (Basso and Bonetto, 2016; Ciardiello et al., 2020). The most frequently employed microvesicle markers are mainly integral membrane proteins and cytoskeletal proteins, including KIF23, RACGAP, CSE1L, ARF6, and EMMPRIN (Muralidharan-Chari et al., 2009; Antonyak et al., 2012; Li et al., 2012; Ghossoub et al., 2014; Greening et al., 2017). Several studies suggest that MVs carry a wide variety of cargo including cell surface receptors, cytosolic signaling proteins, metabolic enzymes, and nuclear proteins indicating their role in intercellular communication (Antonyak et al., 2011; Kreger et al., 2016). Specifically, they contain mRNA, lncRNAs and miRNAs, dsDNAs, cytoplasmic proteins suggesting their potential role in exchanging genetic material between cells (Balaj et al., 2011; Greening et al., 2017). Neural stem cells derived MVs have been shown to influence synaptic activity, nerve protection and regeneration, and neuronal development (Marzesco et al., 2005; Lai and Breakefield, 2012). Studies have also demonstrated the pleiotropic effects of MVs by delivering pluripotent transcription factors (Ratajczak et al., 2006a; Ratajczak et al., 2006b). However, shedding of MVs derived from endothelial cells (Minagar et al., 2001; Schindler et al., 2014), neurons (Bianco et al., 2005; Horstman et al., 2007; Colombo et al., 2012), glial cells (Saijo and Glass, 2011; Colombo et al., 2012; Verderio et al., 2012; Agosta et al., 2014), and platelets (Lee et al., 1993; Geiser et al., 1998) have been reported with regards to incidences of stress, oxygen radicals, inflammation, ischemia, and other stimuli. (Porro et al., 2015). Therefore, spike in MVs would likely be associated with stroke, vascular dementia, inflammatory, and other neurodegenerative diseases (Doeuvre et al., 2009; Porro et al., 2015). For instance, increase in MV production is observed in Alzheimer patients with mild cognitive impairment. These Alzheimer associated MVs are also characterized for their increase in toxicity (Verderio et al., 2012; Garzetti et al., 2014; Joshi et al., 2014). Likewise, the role of oligodendroglioma derived MVs as signal transductor has been shown to trigger neuronal apoptosis and suppressing neuronal sprouting (Porro et al., 2015; D'Agostino et al., 2006). Given that MVs derive from the plasma membrane, they could effectively reflect the intercellular activities in the microenvironment, exhibiting characteristics as prominent markers of the pathophysiology of the CNS (Porro et al., 2015).

#### 2.1.2 Apoptotic bodies

Apoptotic cells also release extracellular particles called apoptotic bodies (Kakarla et al., 2020). These particles are generally described as vesicles that carry nuclear fragments and cellular organelles due to apoptosis (Kakarla et al., 2020). Being one of the largest extracellular particle, their size ranges from 800 to 5000 nm in diameter (Serrano-Heras et al., 2020). Originally these particles were considered cell debris and were often overlooked in studies of circulating vesicles (Kakarla et al., 2020). However, apoptotic bodies purified from neurological patients did not differ in morphology or size distribution from those purified from healthy volunteers (Serrano-Heras et al., 2020). Additionally, they identified neurological apoptotic body biomarkers that were consistent regardless of the health condition of their cell of origin (Serrano-Heras et al., 2020). These studies are not conclusive that these vesicles have a distinct role in pathological conditions; hence, further studies of the functionality of these particles is needed.

#### 2.1.3 Ectosomes, oncosomes, and beyond

Although classification of large vesicles is evolving, ectosomes and large oncosomes have been widely studied (Keerthikumar et al., 2015). Ectosomes are released from the plasma membrane and are implicated to have role in cancer (Keerthikumar et al., 2015; Thery et al., 2018). Large oncosomes are produced from several cancer cells when stimulated involving EGFR and overexpression of membrane-targeted Ak1t (Di Vizio et al., 2012). These vesicles differ in morphology and biogenesis from oncosomes which are any EV subtype that carries oncogenic cargo (Di Vizio et al., 2012). Large Oncosomes are 1,000–10,000 nm in diameter and are derived from ameboid tumor cells (Di Vizio et al., 2012). These cancer cells use amoeboid movement in order to invade other cells through narrow 3 μm-wide microchannels at a fast velocity that cannot be stopped by integrin inhibition (Wu et al., 2021). Additionally, amoeboid cancer cells have been suggested to have drug-resistant properties (Graziani et al., 2022). Considering their highly invasive properties, better ways of targeting them in clinics are needed. Further research should investigate how large oncosomes found in biofluids can be used as a liquid biopsy alternative to detect the presence of ameboid tumors.

### 2.2 Exosomes

Exosomes are nano sized EVPs (∼30–200 nm) of endocytic origin that have demonstrated clinical potential as therapeutic agents given their role in pathogenesis and the biologically active molecules they encapsulate. (Kalluri and LeBleu, 2020; Fan et al., 2022). Exosomes are intraluminal vesicles derived from inward budding of endosomal multivesicular bodies that are released into the extracellular space via exocytosis. (Alvarez-Erviti et al., 2011; Kahlert and Kalluri, 2013; Basso and Bonetto, 2016; van Niel et al., 2018; Jeppesen et al., 2019). The process of sorting exosomal cargo mainly revolves around the endosomal sorting complex (ESCRT), comprised of four complexes (ESCRT-0, I, II, and III) derived from up to thirty protein accessories (Greening et al., 2017; van Niel et al., 2018; Mathieu et al., 2019). Likewise, another major component are the Rab GTPase proteins, largely involved in modulations of intercellular vesicle transportation and budding and release of vesicles (Colombo et al., 2014; Greening et al., 2017; Mathieu et al., 2019; Kalluri and LeBleu, 2020). As a result, common surface markers unique to exosomes include endosome related components like flotillin, CD63, TSG101, and Alix (Meckes et al., 2013; Clark et al., 2015; Costa-Silva et al., 2015; Kowal et al., 2016; Song et al., 2020). Like MVs, exosomes have been found to contain proteins and nucleic acids (Zhang et al., 2019a). However, it has been found that they differ in their lipid, protein, RNA, and DNA composition, emphasizing their potential unique roles (Wei et al., 2017; Pegtel and Gould, 2019).

In the context of brain, exosomes facilitates intercellular communication, synaptic plasticity, neurogenesis, and neuronal stress response by transferring cell type specific coding and non-coding RNAs, miRNAs, proteins, and lipids (Lachenal et al., 2011; Bahrini et al., 2015; Li et al., 2018a; Saeedi et al., 2019). Consequently, there have been reports of pathogenic amyloids and protein deposits found within and outside of brain cells associated with neurodegenerative diseases (Sweeney et al., 2017; Saeedi et al., 2019). Thus, access to biofluids containing exosomes with pathogenic protein aggregates may be capable of profiling the heterogeneity of neurological diseases and disorders via simple and non-invasive liquid biopsy (Lai and Breakefield, 2012; Hornung et al., 2020; Younas et al., 2022).

Researchers have investigated the associations between exosomes and progression of neurodegenerative diseases, including both synucleinopathies (Kunadt et al., 2015; Stuendl et al., 2016) and tauopathies (Saman et al., 2012; Guix et al., 2018), and prion diseases (Bellingham et al., 2012; Chiasserini et al., 2014). Exosomes derived from neurons and glia constitute a sophisticated and interconnected network that influences physiological functions of the CNS (Holm et al., 2018; Fan et al., 2022). This makes them an indispensable component in a series of protective mechanisms of the CNS including angiogenesis (van Balkom et al., 2013; Liang et al., 2016), inhibition of the neural apoptosis (Song et al., 2019), neuroimmune regulation (Kolios and Moodley, 2013; Doeppner et al., 2015), formation of myelin sheath (Boecker et al., 2018; Madison and Robinson, 2019; Yin et al., 2021), growth of axon (Li et al., 2018b; Delpech et al., 2019; Guo et al., 2019; Fan et al., 2022). Nevertheless, in tumor microenvironment or during progression of neurodegenerative diseases, exosomes are linked to distribution of amyloid-β peptides and α-synuclein and tumor metastasis (El Andaloussi et al., 2013; Kalluri and LeBleu, 2020; Fan et al., 2022).

### 2.3 Viruses

The viruses must also be considered as a subset of the vast EVP canopy (Ghosh et al., 2020; Dogra et al., 2021). Many viruses follow the late lysosome or multivesicular body (MVB) followed by exocytosis mechanism for egress (Alem et al., 2021; Dogra et al., 2021; Elmore et al., 2021). As shown by Dogra et al. (2021) the biogenesis of SARS-coV-2 have an egress pathway identical to the exosomes. While, other viruses, such as HIV, pinch off the plasma membrane (Brügger et al., 2006). As a result, all viruses fall under the classification of either microvesicles/ectosomes (pinching off the plasma membrane), exosomes (exocytosis), or other pathways EVP release.

### 2.4 Mitochondrial derived-extracellular vesicles

The mitochondria may function as a communication channel between neurons and provide neuroprotective and neurorecovery functions (Hayakawa et al., 2016). However, it is unknown whether communication is achieved through the secretion of whole mitochondria, mitochondria-encapsulated microvesicles, or EVPs packed with mitochondrial cargo. Researchers have identified vesicles carrying cargo of mitochondrial proteins, lipids, and mtDNA (Miller et al., 2022; Popov, 2022). These vesicles have been potentially misinterpreted as exosomes. Recent studies have identified that these vesicles are not exosomes but rather, a subtype of EVs called mitochondrial derived-vesicles (MDV) (D'Acunzo et al., 2021; Nakamya et al., 2022). It has been described that neurological diseases, including Alzheimer’s, Parkinson’s, substance use disorder and down syndrome, can affect mitochondrial structure and function (D'Acunzo et al., 2023).

Two types of MDVs have been described–mitochondrial derived-intracellular vesicles (MDIV) and mitochondrial derived-extracellular vesicles (MDEV), also known as mitovesicles (D'Acunzo et al., 2021; Nakamya et al., 2022; Heyn et al., 2023). MDIVs are single-layer vesicles that originated from the outer membrane, whereas the MDEVs are bilayer vesicles due to its origin from both the inner and outer membrane (Sugiura et al., 2014; Heyn et al., 2023). Given their difference in biogenesis, electron microscopy is frequently employed to distinguish between MDIV and MDEV (Sugiura et al., 2014; Heyn et al., 2023). Likewise, differences in lipid content that stems from varying EVP biogenesis processes can also distinguish MDVs from other EVPs (D'Acunzo et al., 2021). For example, MDIVs are TOMM20-positive, PDH-negative and MDEVs are TOMM20-negative, PDH-positive (Heyn et al., 2023). Furthermore, MDEVs and MDIVs do not express exosome or microvesicle markers (e.g., Annexin A1, Annexin A2, Alix, TSG101, CD63), thus, implying they belong to their own subtype. Furthermore, recent research has revealed that MDEVs transport distinct cargo in healthy *versus* diseased states, as observed in both humans and rodents with Down Syndrome and Cocaine Use Disorder (D'Acunzo et al., 2021; D'Acunzo et al., 2023). Although the field of MDEVs is still emerging, further research is necessary to comprehend the role of these subtypes in the etiology and diagnosis of mitochondrial abnormalities (D'Acunzo et al., 2021; D'Acunzo et al., 2023). Nevertheless, the current research confirms previous findings of the association between physiological disorders and mitochondrial abnormalities (D'Acunzo et al., 2021; Nakamya et al., 2022; D'Acunzo et al., 2023).

### 2.5 Exomeres

First considered as cellular debris, exomeres (<50 nm), unlike other EVs are non-membranous nanoparticles (Zhang et al., 2018; Zhang et al., 2019b). Since these vesicles are lacking a lipid bilayer and expression of ESCRT, it is suggested that they do not originate from the plasma membrane or endocytic pathway like the other subtypes (Zhang et al., 2019b). Regardless of their small size, they have been detected to contain proteins, lipids, and nucleic acids (Zhang et al., 2018). It is suggested from their protein enrichment that their cargo is closely associated with the endoplasmic reticulum, mitochondria, and cytoskeletal microtubules (Zhang et al., 2019b). *In vitro* studies have shown that exomeres can contain functional cargo that can alter recipient cells (Zhang et al., 2019b). Yet, the biogenesis and the exact function of exomeres remain unknown. Given the ambiguity in their biogenesis, several questions are being raised regarding the formation of additional particles during their rigorous isolation process.

### 2.6 Supermeres

A recent study conducted by Zhang et al., reported the discovery of a new distinct nanoparticle named supermeres (Zhang et al., 2019b; Zhang et al., 2021a). While ultracentrifugation of the supernatant of exomeres at 376,000 × g for 16 h the authors discovered a small pellet (Zhang et al., 2019b). After analyzing this pellet authors found that these nanoparticles were distinct from exosomes in size, morphology, composition, and cellular interactions. As a result, the authors termed the subcategory of nanoparticles supermeres–supernatant of exomeres. These nanoparticles are suggested to have a functional application since they are potentially enriched in clinically relevant proteins that were previously reported in exosomes (e.g., amyloid precursor protein (APP), cellular-mesenchymal-epithelial transition factor (MET), glypican 1 (GPC1), argonaute-2 (AGO2), TGFβ-induced (TGFBI), numerous glycolytic enzymes) and extracellular RNA (exRNA; miR-1246)) (Zhang et al., 2021a). Further studies verifying the existence of these particles are required since there is no clear understanding of how supermeres are formed and what is their function. Ultracentrifugation could lead to the formation of additional particles, and it is not clear whether supermeres are formed during ultracentrifugation or not.

### 2.7 High and low density lipoproteins

Cholesterol containing particles have been comprehensibly studied for their size, biogenesis, and disease pathology, mainly related to cardiovascular (Ishibashi et al., 1993) and Alzheimer’s diseases (Tcw et al., 2022).These particles are classified as very low density lipoproteins (VLDL) (35–200 nm), low-density lipoproteins (LDL) (20–26 nm), and high-density lipoproteins (HDL) (5–8 nm) and other subtypes of them (Claude, 1970; Liangsupree et al., 2021). In a recent study Murillo et al. investigated the exRNA of variable particles and it was shown that HDL and LDL carry distinct RNA, while argonaute proteins and vesicles of different density cargo unique RNA signatures (Murillo et al., 2019). Furthermore, It is important to note that LDL is up taken and released by cells through receptor-mediated endocytosis, a similar process to exosomes secretion (Tcw et al., 2022).

## 3 Advances in isolation technologies for EVPs

Common sources of EVPs include tissue, cell supernatant, and a wide variety of biofluids including blood, saliva, urine, breast milk, etc. (Valadi et al., 2007; Keller et al., 2011; Lässer et al., 2011; Crescitelli et al., 2021). In contrast to tissue and cell supernatant samples, biofluids are more accessible and less invasive. However, samples of bodily fluid generally have larger starting volumes, resulting in challenges associated with dilution or low EVP yield and purity (Ramirez et al., 2018). This issue is further exacerbated when attempting to isolate cell-type specific EVPs or specific EVP subpopulations from biofluids (Su et al., 2017; Antes et al., 2018; van Niel et al., 2018). Therefore, the key feature of a clinically applicable and reliable EVP isolation method would need to address: 1) sensitivity to individual subpopulations of EVPs; 2) purity and throughput of isolated EVPs; 3) reproducibility, standardization, and scalability; 4) external validity when considering various clinical settings and samples used (Younas et al., 2022). Currently, the established isolation methods include differential ultracentrifugation (UC), density gradient ultracentrifugation (UC-DG), size exclusion chromatography (SEC), immunoprecipitation via beads, field-flow fractionation (FFF), tangential flow filtration (TFF), nanofluidic deterministic lateral displacement (nanoDLD), and acoustic trapping technology (Wunsch et al., 2016; Kim et al., 2017; Smith et al., 2018; Liangsupree et al., 2021).

### 3.1 Differential centrifugation (UC), density gradient ultracentrifugation (UC-DG)

UC and UC-DG remain the most frequently implemented method due to its high EVP yields (Royo et al., 2020; Liangsupree et al., 2021). UC utilizes a series of gradually increasing centrifugation to sequentially pellet, remove debris and isolate EVPs in a stepwise manner, allowing for efficient isolation even given a large starting volume (Carnino et al., 2019; Brennan et al., 2020). This method also does not require any additional chemical reagents ensuring the functionality of the EVPs isolated (Liangsupree et al., 2021). Nevertheless, the resulting pellet from UC are expected to cause EVP aggregations and would include a mixture of all EVPs, suggesting lower purity for selected subtypes (Szatanek et al., 2015; Carnino et al., 2019). Individual biofluids’ inherent biochemical compositions also interfere with this isolation process (Ramirez et al., 2018); the polymeric-Tam Horsfall protein, frequently found in urine, tends to bind to EVPs which causes the complex to pellet at lower centrifugation speed, decreasing the overall EVP yield and purity (Wachalska et al., 2016); EVP pellet isolated from breast milk via UC appears to solidify due to higher concentration of whey and casein protein, thus posing great difficulty for EVP resuspension (Ramirez et al., 2018).

In contrast, UC-DG implements sucrose, iohexol, or iodixanol-based density gradient on top of the UC protocol. (Cvjetkovic et al., 2014). This allows for further segregation of the UC isolated EVP pellet based on the individual buoyant density of the subpopulations found within the pellet (Konoshenko et al., 2018; Carnino et al., 2019). Nonetheless, in exchange for the improved segregation, the set up for UC-DG are exceedingly sophisticated and time consuming, requiring up to 2 days for completion (Théry et al., 2009; Konoshenko et al., 2018). Additionally, studies have reported EVP throughput of both UC and UC-DG is significantly dependent on the centrifuge and rotors used (Théry et al., 2006; Momen-Heravi et al., 2013; Witwer et al., 2013; Cvjetkovic et al., 2014; Gardiner et al., 2016; Konoshenko et al., 2018).

### 3.2 Size exclusion chromatography (SEC)

To further simplify the EVP isolation process, SEC-based methods are developed. SEC employs a column filled with porous resin of pre-determined diameter to effectively drain and isolate all EVP and its subpopulations based on their hydrodynamic radius found within the loaded biofluid (Barth et al., 1994). Isolated EVP subtypes are then eluted as separated fractions in the order of decreasing diameters. This mechanism prevents EVP aggregates and preserves their functionality (Gámez-Valero et al., 2016). Most importantly, SEC minimizes the amount of time and effort needed considerably for EVP isolation (Gámez-Valero et al., 2016). This can make or break the clinical applicability of EVP based liquid biopsy. Despite the rigorous segregation by size, SEC offers limited separation of EVP given the overlapping diameters of various subgroups (Carnino et al., 2019; Brennan et al., 2020). In addition, the eluting solutions used for SEC would further dilute the concentration of each EVP fractions (Brennan et al., 2020; Liangsupree et al., 2021).

### 3.3 Immunoprecipitation (IP)

Immunoprecipitation is a fast and simple affinity-based method that targets the surface protein markers of the EVPs via magnetic beads to isolate the selected EVP population. This method is frequently used in conjunction with other isolation methods as a purification step (Carnino et al., 2019). The specificity of the method could be adjusted by attaching specific antibodies to the magnetic beads in correspondence to EVP subtypes or different cell types, thus providing high purity sample output (Tauro et al., 2012). Consequently, the high selectivity of this method is highly contingent on presence of sufficient number of beads and proper optimization of the ligands to allow for maximal binding (Carnino et al., 2019). Thus, this suggests immunoprecipitation would be less efficient in biofluids with complex compositions of varying enzymes and molecules, for instance, plasma, due to binding competition (Hage et al., 1993; Carnino et al., 2019). There is also the need to account for the high cost of beads with specific ligands and difficulties with detachment of antibodies from EVPs for preservation of integrity (Heath et al., 2018).

### 3.4 Field-flow fractionation (FFF) and as.ymmetrical flow field-flow fractionation (AF4)

FFF is a term used to describe a plethora of flow-based separation technique that applies an external perpendicular force on the flow direction of the sample causing accumulation of particles along the bottom wall of the narrow channel (Schallinger et al., 1985). Correspondingly, the counteracting Brownian motion of the particles within the fluid would diffuse, separating the particles into layers based on their diffusion coefficient and allowing for elution at different time point (Zhang and Lyden, 2019). The smaller particles with higher diffusion coefficient would be near the upper layer thus eluting faster than larger particles near the bottom (Sitar et al., 2015). The most frequently used FFF for the purpose of EVP isolation is the asymmetrical flow field-flow fractionation (AF4) that utilizes cross-flow as the external force and replaced the bottom wall with a pre-determined pore size permeable (Zhang and Lyden, 2019). The biggest advantage of AF4 relative to other size-based isolation techniques is its flexibility; the cross-flow could be adjusted between runs to accommodate for fluid samples of varying degrees of particle heterogeneity (Sitar et al., 2015). Likewise, the solvent used in AF4 could also be replaced with PBS or the original EV formulation buffer to preserve EV integrity for functionality experiments. However, similar to SEC, AF4 has the tendency to dilute the samples post isolation. Additionally, AF4 is not catered towards large volume sample isolation as this could likely result in self-association and overloading effects (Liangsupree et al., 2021).

### 3.5 Tangential flow filtration (TFF)

TFF, otherwise known as cross-flow filtration, is an improved size-based filtration technique that implements solvent flowing in the direction tangent to the semi-permeable membrane, effectively preventing the buildup of larger particles and filter cake formation (Busatto et al., 2018; McNamara et al., 2018). The particles smaller than the pores would then travel across the membrane due to the transmembrane pressure (Kim et al., 2021). A combination of multiple membranes of different pore sizes could achieve proper EVP isolation and effectively segregate the subpopulations. Additionally, utilization of TFF is often coupled with other methods to further concentrate the resulting isolated EVPs, providing stronger signals for downstream analysis. This suggests TFF is highly efficient in isolation of EVPs in large volume of diluted samples (Liangsupree et al., 2021). The isolation process also effectively preserves the integrity and biological activity of the EVPs (Jia et al., 2022). Nevertheless, similar to other size-based isolation techniques, TFF has limited separation of EVP subpopulations given overlapping diameters of the subtypes.

### 3.6 Nanofluidic deterministic lateral displacement (nanoDLD)

The nanofluidic device nanoDLD (Smith et al., 2018), is a size-based isolation method that use asymmetric pillar arrays to deflect particles in specific trajectories in accordance with their size (Kim et al., 2017). Smaller particles would exhibit less disruption and would flow through the pillars in a “zigzag” pattern (Kim et al., 2017). Meanwhile, larger particles would likely be disrupted by the pillars, thus, would typically travel in a “bumping” pattern (Wunsch et al., 2016; Kim et al., 2017; Smith et al., 2018). The displacement in the larger particles allows for effective segregation between EVPs of varying hydrodynamic diameter (Wunsch et al., 2016; Kim et al., 2017; Smith et al., 2018; Liangsupree et al., 2021). This mechanism makes high purity particle isolation via nanoDLD extremely efficient and reproducible. However, it requires the use of a silicon chip which significantly limits the amount of sample it could process in a single run (Wunsch et al., 2016). Correspondingly, similar to filtration-based methods, nanoDLD is prone to clogging, therefore, prefiltration of larger particles is required (Liangsupree et al., 2021).

### 3.7 Acoustic trapping

Acoustic trapping technology, on the other hand, heavily relies on ultrasonic wave scattering to effectively cluster and separate EVPs based on their size, density, and compressibility (Rezeli et al., 2016; Bryl-Górecka et al., 2018; Ku et al., 2018; Ku et al., 2019). This method requires addition and retainment of seeding particles, most commonly polystyrene beads, using the acoustic standing wave prior to loading the samples. Once the sample is loaded, the particles within would aspirate and cluster with the seeding particles using the secondary acoustic wave through particle-particle interactions. To retrieve the isolated EVP subtypes, the clusters are washed and released from the beads once the acoustic wave is turned off (Ku et al., 2018). Acoustic trapping has demonstrated high efficacy and strong enrichment performances for sample volume as low as 12.5 ul (Bryl-Górecka et al., 2018; Liangsupree et al., 2021). Nevertheless, the set up and maintenance requires large amount of funding and the device itself are only functional when high power inputs are available (Hammarström et al., 2021).

## 4 Extracellular vesicles and particles in the healthy central nervous system

While many publications have exclusively used the terms “exosomes” or “ectosomes” etc., the isolation methods used in the respective studies do not exclusively separate such particles, but a heterogenous population. For the purpose of inclusion of all particles, in this review, we have used the term EVPs as defined by the International Society of Extracellular Vesicles (ISEV) (Thery et al., 2018).

### 4.1 Oligodendrocytes and neurons

Oligodendrocytes are specialized glial cells that wrap around the axons of neurons forming myelin sheaths in the CNS (Pegtel et al., 2014). The myelination of axons is crucial for proper conduction of impulses (Xiao et al., 2021). During the myelination process axons and oligodendrocytes intercommunicate in order to maintain axon integrity and survival (Pegtel et al., 2014). The relationship is evident in demyelinating diseases where myelin damage is strongly associated with neuronal and axonal degeneration (Xiao et al., 2021). Research suggests that oligodendrocyte-derived EVPs mediate signaling between oligodendrocytes and neurons (Frühbeis et al., 2013; Pegtel et al., 2014; Delpech et al., 2019; Pistono et al., 2021).

Studies suggest that oligo-EVPs are released as a result of neuron activation starting a cascade of events (Delpech et al., 2019). First, glutamatergic signaling from neurons occurs (Delpech et al., 2019). This results in glutamate inducing a Ca2+ influx in oligodendrocytes, which causes the activation of GTPase Rab35 that in turn leads to the release of oligo-EVPs (Lachenal et al., 2011; Delpech et al., 2019) Neurons exposed to oligo-EVPs display increased firing rates as well as altered gene expression. Therefore, the increased activation of neurons increases the release of oligo-EVPs, which then enhances the activation of the neuron, fueling a cyclical relationship (Krämer-Albers, 2020). Hyperactivation of neurons has been associated with neurodegenerative disorders (Xiao et al., 2021). Yet, oligo-EVPs cause an enhancement of neuronal firing without causing excitotoxicity in healthy brains (Krämer-Albers, 2020). In fact, oligo-EVPs may have a neuroprotective role (Krämer-Albers, 2020; Oyarce et al., 2022). A study found that neurons exposed to oligo-EVPs under conditions of oxidative stress and nutrient deprivation had higher metabolic activity than neurons exposed to HEK293T-derived exosomes or artificial liposomes (Frühbeis et al., 2013). Thus, suggesting that oligo-EVPs uniquely aid neuronal health. Further research should be done to understand how the release of oligo-EVPs affects the signaling of other neurotransmitters and its role in regulation and possible clinical application.

The purpose of oligo-EVP uptake may be different between cell types. *In vitro* oligo-EVPs were seen to be taken up by neurons and microglia, and infrequently by astrocytes or oligodendrocytes (Figure 2) (Krämer-Albers, 2020). In order to validate these findings an *in vivo* study was done using transgenic mice and oligo-EVPs carrying Cre-recombinase (Frühbeis et al., 2013). Given the activation of the reporter by Cre-recombinase requires the release of Cre from the endosome and translocation to the nucleus, any expression of Cre would directly correlate to oligo-EVP uptake; increased levels of Cre expression were observed in neurons correspondingly (Frühbeis et al., 2013). However, in the earlier studies microglia were also seen to take up oligo-EVPs by macropinocytosis. (Krämer-Albers, 2020). It is believed that these EVPs are then trafficked to lysosomes for degradation in order to clear oligodendroglial myelin membrane debris through their EVP uptake (Delpech et al., 2019). Therefore, the lack of visualization using the Cre reporter, is most likely due to the degradation of EVP-cargo in the endo-lysosomal system. In neurons, Cre was expressed to demonstrate that oligo-EVPs can be internalized *in vivo* (Frühbeis et al., 2013). Additionally, the number of recombined neurons did not change due to uptake at axonal and soma-dentric sites, indicating both sites may be used (Frühbeis et al., 2013). As a result, it is clear that the cargo of oligo-EVPs can alter gene expression of neurons (Frühbeis et al., 2013). However, since both uptake sites contain receptors that regulate neuronal signaling, research is needed to investigate whether oligo-EVPs alter the membrane of these sites and can activate these receptors.

### 4.2 Microglia and neurons

Microglia are the immune cells of the CNS (Williams et al., 1994; Sousa et al., 2017). To ensure homeostasis is maintained they produce soluble factors that mediate inflammatory responses (e.g., chemokines, cytokines, and free radicals) (Kettenmann et al., 2011; Paolicelli et al., 2019). These biomolecules can be exchanged between cells through the use of microglia-derived extracellular vesicles (microglia-EVPs) in order to communicate support for infection and brain damage (Paolicelli et al., 2019). Additionally, microglia-EVPs can signal neuronal activation through surface components (Ceccarelli et al., 2021; Picciolini et al., 2021). While neuroinflammation aims to solve an injury and restore brain homeostasis, the dysregulation of such responses can become unfavorable and even neurotoxic (Brites and Fernandes, 2015). Through the study of microglia-EVPs we can gain a deeper understanding of how healthy conditions are maintained.

Microglia-EVPs have also been suggested to play a role in neurite outgrowth, modulating neuronal activity, and orchestrating innate immunity (Delpech et al., 2019). The surface components of microglia-EVPs, rather than their cargo content, have been found to have a direct effect on neurotransmission, causing an increase in miniature excitatory postsynaptic current (mEPSC) (Delpech et al., 2019; Ceccarelli et al., 2021). Specifically, microglia-EVPs enrich ceramide and sphingosine production in neurons and increase synaptic activity by facilitating SNARE and synaptic vesicle release (Delpech et al., 2019; Aires et al., 2021). Further research is needed to understand how microglia-EVPs supportive role in synaptic vesicle release may be dysregulated in psychiatric chemical imbalances. Microglia-EVPs have also been shown to alter neurotransmission by transporting hydrophobic ligands on their surface (Paolicelli et al., 2019; Ceccarelli et al., 2021). For example, it was found that microglia-EVPs carry endocannabinoid N-arachidonoylethanolamine (AEA) on their surface which can be used to activate type-1 cannabinoid receptors (CB1) (Gabrielli et al., 2015a; Gabrielli et al., 2015b; Paolicelli et al., 2019). The activation of CB1 receptors on GABAergic neurons leads to the inhibition of presynaptic transmission (Manzoni and Bockaert, 2001; Mackie, 2006). A variety of neurological activities, such as mood, memory, and cognition, are mediated by CB1 receptors (Kendall and Yudowski, 2016; Franzen et al., 2022). Thus, altered expressions of the CB1 receptors have been observed in various neurodegenerative diseases, such as Alzheimer’s disease, Parkinson’s disease, and Huntington’s disease (Bisogno and Di Marzo, 2010; Vasincu et al., 2022). These alterations may be associated with microglia-EVPs (Aires et al., 2021). Correspondingly, the communication between microglia-EVPs and neurons are bidirectional; microglia-EVPs are capable of being able to activate neurons and vice versa (Figure 2) (Albertini et al., 2020). When serotonergic signaling occurs, 5-HT is released from neurons, binding to 5-HT receptors on microglia, and releasing EVPs (Glebov et al., 2015; Albertini et al., 2020). Depression which has been associated with low levels of serotonin has also been associated with neuroinflammation (Brites and Fernandes, 2015; Cowen and Browning, 2015). A deficiency of serotonin may lead to modification of microglia-EVPs, which in turn suppresses the neuroimmune system. However, this association needs to be further investigated.

### 4.3 Astrocytes and neurons

Astrocytes are known to play important roles in various neurological processes, including the support and maintenance of neurons, the regulation of brain blood flow, and the formation and repair of the blood-brain barrier (Sidoryk-Wegrzynowicz et al., 2011; Gharbi et al., 2020). There is increasing evidence that astrocyte-derived EVPs (astro-EVPs) may be involved in a number of neurological processes, including the modulation of inflammation, the promotion of neurogenesis, and the repair of damaged tissue (Figure 2) (Gharbi et al., 2020; Upadhya et al., 2020). In addition, astro-EVPs appear to regulate the concentration of neurotransmitters (Upadhya et al., 2020; You et al., 2020). Astro-EVPs’ cargo could be further investigated to understand better their neuroprotective mechanisms.

Upon changes in the environment, astrocytes increase EVP release and modify EVP cargo in order to maintain homeostasis (Gharbi et al., 2020). Jovicic et al. suggests that miRNAs contained in EVPs secreted by mouse astrocytes differed from those present in the cell of origin (Jovicic and Gitler, 2017). According to these findings, astrocytes select specific miRNAs for EVP transport in correspondence to varying functions (Upadhya et al., 2020). For example, when in an environment with cytokines IL-1β and TNF-α, astro-EVPs will contain miRNAs that support neuronal function (Upadhya et al., 2020). Like miR-125a-5p and miR-16-5p which activate the kinase receptor for neurotrophin 3 thereby promoting neuronal survival and differentiation (Upadhya et al., 2020). In stressful conditions, astro-EVPs have been found to carry cytoprotective heat shock protein 70 (HSP70) (Guha et al., 2019a; Upadhya et al., 2020). Whereas, in conditions of hypoxia and hypoglycemia, astro-EVPs have been found to carry functional prion proteins (Guitart et al., 2016; Upadhya et al., 2020). According to this study, these EVPs are involved in activating specific signaling pathways that enhance neuronal protection (Upadhya et al., 2020).

Neurotransmitter regulation is a complex process that is essential for maintaining normal brain function (Borodinsky et al., 2014). In order to regulate the concentration of glutamate, astrocytes utilize excitatory amino-acid transporter (EAATs) proteins on their surface (Murphy-Royal et al., 2017; Mahmoud et al., 2019; Todd and Hardingham, 2020). These transporter proteins have also been found in astro-EVPs indicating they may maintain neuronal homeostasis by reducing excitotoxicity in the brain, often associated with numerous neuropathological conditions (Upadhya et al., 2020). Further research is needed for therapeutic applications of astro-EVPs containing EAAT.

## 5 Extracellular particles in the pathological central nervous system

### 5.1 Neurodegenerative diseases and aging

There is evidence that EVPs may play a role in the development and progression of neurodegenerative diseases, which are conditions characterized by the progressive loss of function and death of neurons in the brain and nervous system (Thompson et al., 2016). These diseases include conditions such as Alzheimer’s disease (AD), and Parkinson’s disease (PD). Aging is a major risk factor for the development of neurodegenerative diseases, and it has been suggested that the aging process itself may be influenced by EVPs (Robbins, 2017; Hou et al., 2019).

Since accelerated aging may lead to neurodegeneration and other physical declines (Hou et al., 2019), biomarkers may offer a means to monitor aging and ensure that preventative care is provided. It is suggested that mitochondrial abnormalities occur with age and neurodegeneration (Lazo et al., 2021). Scientists analyzed EVP mtDNA from individuals aged 30–64 using both cross-sectional and longitudinal methods to determine whether human mtDNA levels vary with age (Lazo et al., 2021). They discovered the inverse relationship between the levels of EVP-associated mtDNA and aging (Chomyn and Attardi, 2003; Lazo et al., 2021). Thus, monitoring the aging process may be possible through the decline in mtDNA levels. Likewise, both AD and PD are associated with mitochondrial abnormalities, making mtDNA in EVPs a valuable insight into these diseases (Bose and Beal, 2016; Perez Ortiz and Swerdlow, 2019; Nakamya et al., 2022). In PD, mitochondrial dysfunction can increase oxidative stress, disrupt cellular material trafficking, impair electron transport chain function, cause calcium imbalances, and disrupt mitophagy (Nakamya et al., 2022). There is interesting evidence suggesting mitophagy is initiated through the PINK1–Parkin pathway, mutations of which are linked to the early onset of recessive Parkinson’s disease (Nakamya et al., 2022). In AD, toxicity associated with Aβ aggregates has been reported to damage mitochondria and cause mitochondrial dysfunction (Reddy and Beal, 2008; Kim et al., 2020). Isolated brain EVPs exposed to Aβ aggregates and H_2_O_2_ have been found to contain mitochondrial structures, RNA and proteins (Kim et al., 2020). This suggests that EVPs may deliver toxic mitochondrial components from damaged mitochondria, promoting cellular pathologies and AD (Kim et al., 2020). A comparison of mitochondrial cargo within EVPs derived from neurodegenerative patients with healthy samples is necessary to determine whether these abnormalities can serve as biomarkers. Further, the mitochondrial cargo of EVPs from aging individuals should also be examined for neurodegenerative-associated components.

Changes in the microenvironment have been linked to neurodegeneration (Delpech et al., 2019; Aires et al., 2021). For example, when midbrain cultures are incubated with IFN-γ and LPS, there is an increase in EVP release and activation of microglia inducing dopaminergic degeneration associated with PD (Aires et al., 2021). It has been suggested that miR-34a carried by astro-EVPs may enter dopaminergic neurons and target the anti-apoptotic Bcl-2 protein, which may also aid the progression of PD (Upadhya et al., 2020). Both microglia-EVPs and astro-EVPs have also been implicated in the pathogenesis of AD (Upadhya et al., 2020; Aires et al., 2021). The activation of P2X7R in microglial cells has been linked to the release of EVPs (Ruan et al., 2020). There is both cognitive improvement and EVP release hindrance when P2X7R is inhibited, emphasizing that EVPs are involved in the promotion of AD (Ruan et al., 2020). Astro-EVPs have been identified as carriers of aggregated proteins such as amyloid beta oligomers (AβO) and protofibrils (Aβ) in AD (Upadhya et al., 2020; Gomes et al., 2022). EVPs isolated from the CSF and blood of patients with AD and PD have been found to contain protein aggregates of Aβ, tau, and α-synuclein (Joshi et al., 2014; Guix et al., 2018; Cicognola et al., 2019; Niu et al., 2020; Upadhya et al., 2020; Li et al., 2022). Thus, it has been suggested that the release of neurotoxic proteins in EVPs allows cells to deliver the protein aggregates to other cells with a higher degradation capacity (Upadhya et al., 2020).

The presence of neurodegenerative proteins in EVPs has led researchers to consider their use as liquid biopsy (Abdel-Haq, 2020). A number of small-scale studies have been conducted, but in order for the results to be valid, larger studies are needed (Kapogiannis et al., 2015). In order to meet this need researchers collected 887 plasma samples from 128 individuals who eventually developed AD and 222 matched healthy controls (Kapogiannis et al., 2019a). They used neuron-EVP biomarker data from these samples which were collected up to 9 years before the onset of AD symptoms, to build a model that could accurately predict future AD diagnoses (Kapogiannis et al., 2019a). Surprisingly, the study found that levels of Aβ42, previously linked to neurotoxicity and neurodegeneration (Upadhya et al., 2020), were not significantly different between individuals with future AD and healthy controls (Kapogiannis et al., 2019a). Validating earlier findings the model did include tau biomarkers (Upadhya et al., 2020) and insulin-receptor-substrate-1 (IRS-1) phosphorylation as predictors, with pSer312-IRS-1 and pY-IRS-1 being powerful individual predictors (Bomfim et al., 2012; Kapogiannis et al., 2015; Mullins et al., 2017; Kapogiannis et al., 2019a; Akhtar and Sah, 2020). These findings support the use of neuron-derived EVP biomarkers as a potential tool for early diagnosis and treatment of AD, and further development as a clinical blood test for the disease is warranted.

### 5.2 Drug addiction

The cycle of substance abuse refers to the repeating pattern of drug use and negative consequences that often occurs in individuals with substance (Feltenstein and See, 2008; Koob and Volkow, 2016). This initial use can then lead to a dependence on the drug, which can cause physical and psychological symptoms of withdrawal when the drug is not taken, reenforcing the need for the drug (Baker et al., 2004; Koob and Volkow, 2016). EVPs have been used to understand the physiological conditions at each stage of the cycle (Chivero et al., 2021).

New insights emphasis that EVP modulated astroglia dysfunction and activation of CB1 enhance dopamine release, thereby contributing to cocaine addiction (Nakamura et al., 2019; Jarvis et al., 2020). In an *in vivo* cocaine addiction model, it was discovered that cocaine reduced the internalization of neuron-EVPs into astrocytes, resulting in less miR-124-3p delivered and decreased glutamate transporter-1 (GLT1) and GFAP levels (Jarvis et al., 2020). A decrease in GLT1 expression may inhibit glutamate reuptake, resulting in greater excitatory response and an increase in dopamine release, furthering addiction (Scofield and Kalivas, 2014; Jarvis et al., 2020). Additionally, the reduced expression of GLT1 and GFAP has also been implicated in several psychiatric disorders, such as anxiety (Jarvis et al., 2020; Jia et al., 2020). This change in EVP cargo may explain the correlation between withdrawal and psychological symptoms. Likewise, both in cocaine treated mice and cells, it has been seen that there is a stimulation of EVP release in the ventral tegmental area (VTA) (Nakamura et al., 2019). These EVPs are suggested to carry 2-Arachidonoylglycerol (2-AG) that binds to CB1, inhibiting GABA release and enhancing dopamine release to promote cocaine dependence (Covey et al., 2016; Nakamura et al., 2019).

As a result of tolerance to opioids, larger doses of the drug may be needed to achieve the same effects, increasing the likelihood of overdose (Chang et al., 2007; Boyer, 2012). Drug resistance and morphine tolerance appear to be linked to upregulation of sonic hedgehog (SHH) proteins in morphine-stimulated astro-EVPs activating SHH signaling in astrocytes via primary cilia (Huang, 2021; Ma et al., 2021; Luxmi and King, 2022). Researchers found that inhibiting either EVP release or primary cilia decreased morphine tolerance indicating its potential therapeutic role (Ma et al., 2021). The choroid plexus (ChP) and CSF has been associated with SHH (Huang et al., 2009; Li et al., 2016; Yang et al., 2021). Since astrocytes can take up ChP-EVPs, this mechanism may also have a role in mediating drug tolerance (Su and Pasternak, 2013; Pauwels et al., 2022). The relationship between neurological and psychiatric disorders with ChP is currently being studied including its role in opioid tolerance and nicotine addiction (Su and Pasternak, 2013; Ochoa et al., 2015; Lallai et al., 2019; Kenny et al., 2022). Additionally, the intercommunication between the ChP and astrocytes mediated by EVPs may contribute to the widespread ChP contamination in the sampling and profiling of brain tissue (Olney et al., 2022).

Research has shown that EVPs can serve as an effective tool for both screening and monitoring individuals with substance abuse disorders (Nakamura et al., 2019; Doncheck et al., 2020; Odegaard et al., 2020; Zhang et al., 2021b; Chen et al., 2022b; Odegaard et al., 2022). For example, a human study utilized plasma EVPs to monitor withdrawal syndrome of heroin and methamphetamine users at 3-month and 12-month stages (Chand et al., 2021; Chen et al., 2022b). Recent studies have found that the expression of EVP miRNA signatures reflects the differences in time point (Li et al., 2018c; Chen et al., 2022b). These EVP miRNAs may be revealed to contribute to psychiatric symptoms if validated by future studies (Chand et al., 2021; Sil et al., 2021; Chen et al., 2022b). Additionally, this data was used to predict the substance patients were using (Chen et al., 2022b). For example, miRNA signature hsa-mia-451a can be used to identify heroin-dependent patients, whereas hsa-mir-21a can be used to identify methamphetamine-dependent patients (Chen et al., 2022b). Another recent and comprehensive study aimed to delineate the role of an extracellular vesicle-associated microRNA-29a in chronic methamphetamine use disorder (Chand et al., 2021). Similarly, using serum EVPs, a team of researchers has successfully distinguished rats dependent on methamphetamine and from those dependent on ketamine (Li et al., 2018c). The unique miRNA profiles of each group could potentially be strongly linked to drug addiction and may help to elucidate the distinct addiction processes involved (Li et al., 2018c). Moreover, another recent study demonstrated the successful use of EVPs to monitor the synaptic genesis of fetuses that were exposed to oxycodone *in utero* in a rodent model (Odegaard et al., 2022). These results underscore the potential use of EVs as a tool for both screening and monitoring in the future.

It has been shown in *postmortem* studies that high levels of hyperphosphorylated tau correlate with the activation of microglia in opiate abusers, suggesting an accelerated AD progression (Kovacs et al., 2015). An investigation of morphine-dependent rhesus macaques provided insight into how EVPs may play a role in mediating this effect (Sil et al., 2021). In the presence of morphine, HIF-1α is seen as a regulator of BACE1 expression–an enzyme essential for the generation of β-amyloid–leading to neuroinflammation (Sil et al., 2021). This inflammation causes the release of amyloid cargo via astro-EVPs, which can lead to the production of Tau (Sil et al., 2021; Gomes et al., 2022). As an indication of the link between neurodegeneration and long-term oxycodone abuse, EVPs isolated from the plasma of non-human primates exposed to the drug were found to contain neurodegenerative and pro-inflammatory biomarkers (Kumar et al., 2021). These biomarkers have a potential clinical application as a risk monitoring tool (Kumar et al., 2021). Furthermore, a study using astro-derived EVPs from morphine-stimulated rodents found that miR-138 activates toll-like receptor 7 (TLR7) leading to neuroinflammation through microglia activation (Liao et al., 2020a). Since neuroinflammation is associated with the risk of developing AD, those who need to take pain relievers long-term may find benefit from silencing TLR7 through EVPs (Liao et al., 2020a).

In conclusion, EVPs have emerged as promising tools for understanding the complex mechanisms of substance abuse and addiction. The cargo of these EVPs, including miRNAs and other biomarkers, have been found to play a critical role in various stages of drug addiction, from initiation to withdrawal symptoms and the risk of developing neurodegenerative disorders (Nakamura et al., 2019; Doncheck et al., 2020; Odegaard et al., 2020; Zhang et al., 2021b; Chen et al., 2022b; Odegaard et al., 2022). Moreover, the use of EVs as a screening and monitoring tool may offer significant clinical benefits, for instance early identification of drug dependence and personalized therapeutic interventions. As further research is conducted in this area, the potential of EVPs to contribute to a better understanding of substance abuse and addiction continues to expand.

### 5.3 Major depression disorder

The most common neuropsychiatric disorder in the general population is Major Depressive Disorder (MDD) which is associated with functional impairment, morbidity, and low quality of life (Ferrari et al., 2013). It is currently being explored whether EVPs can detect drug response for MDD. A larger study was done with 60 participant to investigate miRNA cargo of MDD neuron-EVPs for drug response, indicating that they are reflective of the performance of antidepressant drug post treatment (Saeedi et al., 2021). Further research is need to determine whether miRNA signatures can forecast drug response prior to prescription. Additionally, miR-139-5p found in blood EVPs may play a role in the pathogenesis of depression, as demonstrated by the depressive-like behavior exhibited by mice following the transplantation of blood exosomes from MDD patients (Wei et al., 2020). Therefore, emphasizing its promising use in the diagnosis and treatment of MDD. A study discovered that MDD patients have higher levels of Neuron-EVPs compared to healthy controls (Nasca et al., 2021). The release of these EVPs has been linked to an increase in glutamate levels, which emphasizes the relationship between MDD and abnormal glutamatergic neurotransmission (Frye et al., 2007; Deschwanden et al., 2011; Lachenal et al., 2011; Lee et al., 2013). The study also found that Neuron-EVPs have a higher concentration of insulin receptor substrate-1 (IRS-1) associated with suicidality and anhedonia, confirming previous research linking MDD to insulin resistance, which also impacts glutamate levels (Grillo et al., 2011; Al-Hakeim et al., 2018; Hamer et al., 2019; Nasca et al., 2021). Further research is needed to understand how EVPs modulate glutamate levels in MDD patients, as well as how this knowledge can relate to new treatment methods targeting glutamate pathways like ketamine (Lener et al., 2017; Murrough et al., 2017). In addition to neuron-EVPs, astro-EVPs are also being studied in relation to MDD (Xie et al., 2022). A study found that MDD patients have an increase of inflammatory markers in Astro-EVPs when compared to healthy controls, supporting previous research linking depression to neuroinflammation (Brites and Fernandes, 2015; Troubat et al., 2021; Xie et al., 2022). These studies highlights the potential of EVPs to provide new understanding of the living brain and MDD.

### 5.4 Bipolar disorder

Bipolar disorder (BD) is a mental illness that affects about 1%–5% of the population that involves recurrent episodes of mania, hypomania, and depression and is also associated with a high risk of sucide (Dome et al., 2019). Selective serotonin reuptake inhibitors (SSRIs) often triggering a manic episode (Gitlin, 2019) in patients suffering from BD-related depression, indicating the need for alternative treatment. Through analyzing the content of neuron-EVPs, researchers have proven that infliximab (TNF blocker) can treat BD anhedonia by reducing neuroinflammation (Lee et al., 2021; Mansur et al., 2021). In addition to reducing anhedonia, infliximab treated patient derived-EVPs are associated with insulin cascades in neurons, indicating insulin as a relevant potential target for BD intervention (Mansur et al., 2021). Likewise, by analyzing blood-derived neuron-EVP metabolites a study found a connection between glucose metabolism dysfunction and BD (Du et al., 2022). These metabolites can potentially be used to classify samples from patients with BD, SCZ, MDD and healthy subjects, improving current psychotic diagnosis (Singh and Rajput, 2006). Similarly, research is being done on using miRNA signatures as biomarkers for BD diagnosis (Ceylan et al., 2020). For example, plasma EVPs were used to compare patients in depressive, manic, and euthymic states against a healthy control (Ceylan et al., 2020). 13 miRNAs showed significant differences between patients with BD and healthy individuals, with no significant alterations among different states of BD (Ceylan et al., 2020). Additional studies focusing on neuron-EVPs are necessary to identify potential biomarkers for detecting the transition from a depressive episode to a manic episode and *vice versa*, which can lead to improved treatment options.

### 5.5 Schizophrenia

Schizophrenia (SCZ) is a chronic incurable mental disorder characterized by abnormal thought processes and behaviors, such as delusions and hallucinations (Du et al., 2019). In order to better understand the epistemology, potential biomarkers, and treatments of SCZ, EVPs are employed in SCZ research (Oraki Kohshour et al., 2022). Previously, insulin sensitization was believed to be a side effect of SCZ prescriptions, but recent studies suggest this may be due to excitotoxicity caused by the disorder (Plitman et al., 2014; Kapogiannis et al., 2019b; Pomytkin et al., 2019). As a result of studying peripheral blood neuron-EVPs, scientists were able to determine that insulin resistance exists *in vivo*, even in the absence of drug treatment, emphasizing its role in the dysfunction of brain development and maturation in SCZ (Kapogiannis et al., 2019b; Wijtenburg et al., 2019). To understand the pathophysiology of SCZ and identify potential biomarkers, the first genome-wide miRNA expression profiling in serum-derived EVPs from SCZ patients was performed (Du et al., 2019). The signatures were found to be enriched for genes associated with protein glycosylation, neurotransmitter receptors, and dendrite spine development (Du et al., 2019). Furthermore, hsa-miR-206, a regulator of neutrophic factor expression, was found to be upregulated in blood EVPs of SCZ patients (Du et al., 2019). This data was used to find 11 miRNA signatures that were highly accurate in predicting SCZ and could serve as biomarkers (Du et al., 2019). A follow-up study identified 25 metabolites from neuron-EVPs that can be used to classify samples from patients and controls accurately (Du et al., 2021). These metabolites were enriched in SCZ pathways, such as glycerophospholipid metabolism (Du et al., 2021). These findings point to an important role for EVP metabolite dysregulation in SCZ pathophysiology and indicate a strong potential for their use in SCZ diagnosis. EVPs have also demonstrated treatment potentials. In one study, mesenchymal stem cell-derived EVPs were administered to a rodent model of SCZ, reducing SCZ-like behaviors and neurotoxic levels of glutamate (Tsivion-Visbord et al., 2020). Overall, research focusing on EVPs has contributed to our comprehension of the pathophysiology, potential biomarkers, and treatments of schizophrenia, underscoring the need for follow up studies to enhance diagnosis and management.

### 5.6 Viral infections

There is evidence that EVPs may play a role in the infection and replication of neuronal viruses (Kutchy et al., 2020). For example, research has shown that EVPs can transfer viral particles and genetic material to target cells, potentially contributing to the spread of infection (Kutchy et al., 2020). The role of EVPs in the infection process is greater emphasized in research being done on HIV (Hu et al., 2016; Dagur et al., 2020; Hu et al., 2020). HIV-associated neurocognitive disorders (HAND) are a group of conditions that can affect the nervous, even if there is no detectable viral load (Campbell and Mocchetti, 2021). While neurons are less susceptible to direct infection, infected microglia can contribute to neuronal damage through the release of EVPs (Kannan et al., 2022) One study demonstrates that HIV protein Tat can cause the release of EVPs from microglia, which carry proinflammatory protein NLRP3 cargo (Kannan et al., 2022). When these microglia-EVPs are taken up by neurons, they can lead to synaptodendritic injury and functional impairment, as indicated by decreased mEPSCs (Campbell and Mocchetti, 2021; Kannan et al., 2022). Additionally, Tat has been found to alter the EVP cargo from astrocytes, resulting in impairment of the synaptic architecture of neurons (Kannan et al., 2022). These findings suggest that EVPs may play a mediating role in the microenvironment in the development of HAND (Guha et al., 2019b; Kannan et al., 2022).

### 5.7 Brain tumors

There are various types of brain cancers including glioma, meningioma, astrocytoma, and metastatic brain cancer (Passiglia et al., 2018; Boire et al., 2020). The most well studied of these cancers is a type of glioma referred to as glioblastoma (GBM) (Tominaga et al., 2015; Hallal et al., 2020; Ricklefs et al., 2020; Rana et al., 2021a; Rana et al., 2021b; Ricklefs et al., 2022). GBM is a type of brain cancer that is aggressive and treatment-resistant (Simon et al., 2020). It is characterized by the presence of cancer stem cells (CSCs) and a complex and dynamic microenvironment that includes endothelial cells, astrocytes, and immune cells (Yekula et al., 2020). In the context of GBM, EVPs have been shown to contribute to the maintenance and survival of CSCs and promote GBM recurrence (Simon et al., 2020). Understanding the mechanisms by which EVPs are secreted and target recipient cells in the GBM microenvironment may provide new insights into the disease. For example, GBM-EVPs have been shown to alter the phenotype of normal astrocytes to acquire tumor-supporting capabilities (Zeng et al., 2020; Nieland et al., 2021; Zhou et al., 2022). One study found that EVPs containing miR-19a, delivered from astrocytes to tumor cells, could downregulate PTEN expression, thus upregulating brain metastasis (Simon et al., 2020). Similarly, GBM-EVPs are suggested to stimulate tumor-promoting M2 phenotypes in microglia, as opposed to immune-supporting M1 phenotypes (Simon et al., 2020). GBM-EVPs may also serve as biomarkers for diagnosis, prognosis, and therapeutic response and may be used as a means of drug delivery to the target site (Hallal et al., 2019). For example, a study using serum-derived EVPs found that 7 miRNA signatures can distinguish GBM samples from healthy controls (Ebrahimkhani et al., 2018). Furthermore, miRNA signature miR-9 was found to be upregulated in Temozolomide drug-resistant cells (Simon et al., 2020). In order to reverse this effect, scientists delivered anti-miR-9 to cells via EVP delivery (Simon et al., 2020). As a result of their study, anti-miRNA appears to be a promising therapeutic to counteract these outcomes (Simon et al., 2020). While EVP application for GBM has been extensively researched, EVPs are also showing potential as diagnostic tools for other brain cancers and could aid as a medium to better understand their etiology (Tominaga et al., 2015; Hallal et al., 2020; Ricklefs et al., 2020; Rana et al., 2021a; Rana et al., 2021b; Ricklefs et al., 2022). For example, a study examining brain metastatic cancer has indicated that EVPs containing miRNA-181-c might have the ability to disrupt the blood brain barrier (BBB), thereby facilitating cancer metastasis (Tominaga et al., 2015). Likewise, Graziano et al. has detected varying levels of miR-1, miR-206, miR-663 in the blood EVs of meningioma grade II patients in accordance to patient conditions pre- and post-surgery (Graziano et al., 2021). By conducting research on all types of brain cancer using EVPs, we can gain a deeper understanding of cancer’s underlying mechanisms and develop more effective treatment strategies.

### 5.8 Prion disorders

Prions are misfolded proteins that are transmissible and causative agents for neurodegenerative diseases (Prusiner et al., 1998). Prions are found on the surface of many cells and have been discovered to be associated with EVPs (Bellingham et al., 2012; Cheng et al., 2018). The precise mechanism of prion infections remain a matter of intense debate and EVPs are implicated to have a role in prion infection (Fevrier et al., 2004).

## 6 Clinical application

### 6.1 Liquid biopsy

Cerebrospinal fluid (CSF) biomarkers have been used for the diagnosis of neurodegenerative diseases, but the invasive nature of lumbar puncture collection makes it an impractical choice for routine screening (Wright et al., 2012). Instead, blood is the preferred biofluid for these tests because it can be easily and routinely obtained from patients. However, blood is in contact with the entire body, making it difficult to isolate brain-derived EVPs from those of other tissues (Monteiro-Reis et al., 2021). This introduce the need for CSF biomarkers that can be found in blood (Alawode et al., 2021; Mankhong et al., 2022). Blood-based EVP biomarkers have several advantages as diagnostic tools (Wang et al., 2017). They can be collected using a minimally-invasive procedure and can be repeatedly sampled to monitor changes in the molecular landscape of a disease or treatment outcome (Wang et al., 2017). In addition, this approach may uncover underlying pathological mechanisms that were previously unnoticed (Monteiro-Reis et al., 2021). There are some limitations to this approach, including issues with the isolation and purification of EVPs and the fact that some biomarkers, including miRNA, may change throughout different stages of a disease (Abdel-Haq, 2020). These problems, however, may be alleviated by further research. Many studies have investigated miRNAs, proteins, and metabolites that are useful in diagnosing psychiatric and neurodegenerative disorders (see Table 1). The use of blood-based EVP biomarkers as diagnostic tools in neurological diseases has the potential to enable early detection and treatment, as well as to allow for the monitoring of treatment outcomes and disease progression (Abdel-Haq, 2020).

### 6.2 Therapeutics

Currently, the treatment options for psychiatric and neurological disorders are often limited and may not be effective for all individuals (Howes et al., 2022; Miller and Raison, 2023). In fact, the only approved disease-modifying medication for AD, Aducanumab, has faced controversy in its approval and the minimal data they do have on its effectiveness is limited to those with early onset AD (Howard and Liu, 2020; Beshir et al., 2022). In addition, some therapies can have significant side effects, which potentially reduce significantly quality of life (McCammon and Sive, 2015; Miller and Raison, 2023). For example, many of those with schizophrenia, bipolar disorder, and personality disorders are prescribed antipsychotics which have severe, unpleasant and even lethal side effects including tardive dyskinesia, neuroleptic malignant syndrome, weight gain, diabetes, sedation, emotional blunting and even sudden cardiac death (Moncrieff et al., 2020). It is due to the mental and behavioral changes that many people do not feel like themselves on their medications and avoid taking them (Moncrieff et al., 2020). This highlights the need for therapies with higher sensitivity, higher accuracy, and with no or fewer side effects.

When developing new therapeutics it is important that the drug is selective, does not create an immune response or cause toxicity, and is effective (Strovel et al., 2004; Huggins et al., 2012; Elsharkasy et al., 2020). Recent research has shown that EVPs may be cable of meeting these criteria. The emerging role of EVPs as a therapeutic has been greater understood through studies done on routes of administration, types of cargo that can be delivered, and their inherent nature (Mulcahy et al., 2014; Escude Martinez de Castilla et al., 2021).

According to studies conducted on ways of administration, EVPs enable molecules to cross the BBB, which would not have been possible without them (Qu et al., 2018). Due to the fact that dopamine cannot cross the BBB, scientist examined the effects of intravenously administered dopamine-loaded blood EVPs *versus* administration of free-dopamine on mice (Cestelli et al., 2001). In comparison with those given free-dopamine after 6 h, those given dopamine-loaded blood EVPs had a 15-fold higher distribution of dopamine in the brain (Qu et al., 2018). Additionally, there was no sign of toxicity in the hippocampus, liver, spleen, and lung and there was a minimal immune response (Qu et al., 2018). Other routes of administration have also been investigated. It has been found that intrathecal administration leads to the highest concentration of the drug reaching the brain; however, this route is invasive and not practical for drugs that need multiple doses (Gratpain et al., 2021). Accordingly, researchers are investigating the intranasal route as a second-best route (Gratpain et al., 2021). Since this route goes directly to the brain it has been considered efficient (Liao et al., 2020b; Gratpain et al., 2021). For example, a study administering IFNγ-dendrictic cell-EVPs found the intranasal route targeted the CNS better than the intravenous route and also lead to less accumulation in the liver (Pusic et al., 2021). Additionally, the EVPs have been found to protect the drug from being metabolized by nasal mucosa enzymes (Gratpain et al., 2021). Yet, there is a limitation of how much fluid can be administered before it is drained into the esophagu (Gratpain et al., 2021).

Due to the fact that EVPs are selectively absorbed, they have the potential to be used as targeted drugs. For example, research done on administering IFNγ-dendrictic cell-EVPs intranasally suggest that these EVPs were preferentially taken up by oligodendrocytes, indicating that dendritic cell-EVPs maybe used to target oligodendrocytes (Pusic et al., 2021). It is suggested that the selectivity of EVPs is due to proteins on their surface (Liu et al., 2015). Accordingly, researchers are investigating how these membranes can be edited to be more target (Liu et al., 2015). For example, scientists found that EVPs expressing the neuron-specific rabies viral glycoprotein (RVG) peptide on the membrane could successfully enter cells expressing the acetylcholine receptor on their membranes and not those without it (Liu et al., 2015). Thus, suggesting ways to target specific neurons within the CNS. Likewise, scientists have been striving to develop glioma-targeting EVPs to transport therapeutics exclusively to cancerous cells (Jia et al., 2018; Lino et al., 2021). For example, one study demonstrated how engineered EVPs, containing therapeutic siRNA, could target glioma cells by expressing a protein that binds to neuropilin-1 (NRP-1), which is recognized for its overexpression on the surface of these cells (Jia et al., 2018). This marks a significant shift in cancer therapy from the conventional use of chemotherapy that tends to target all cells (Rébé and Ghiringhelli, 2015). In spite of this, damage to the structure can occur during membrane modification, making large-scale use of these techniques difficult (Herrmann et al., 2021). Therefore, finding EVPs that naturally target specific cells may be a more practical solution.

Although EVPs can be used to administer a variety of cargo including miRNA, proteins, and molecules, a myriad studies have been focusing on their delivery of siRNA. One study administering mu-opioid receptor (MOR) siRNA-loaded EVPs to mice have uncover its ability to prevent morphine relapse (Liu et al., 2015). In another study, EVPs loaded with siRNA targeting Huntingtin mRNA were effectively internalized by mouse primary cortical neurons and significantly silenced Huntingtin mRNA and proteins, further emphasizing the use of siRNA-loaded EVPs for knockdowns (Didiot et al., 2016). Neurological research often employs stereotaxic surgery to infect rodents with viruses that will lead to gene knockdowns. (Nectow and Nestler, 2020). However, this approach is not translational in the human model due to its invasiveness and potential immune response to the virus (Cetin et al., 2006; Sack and Herzog, 2009). It is clear from these earlier studies, that through the use of EVP-loaded siRNA, these knockdowns can still be achieved while also having the potential for therapeutic application in the future.

## 7 Conclusion and future directions

Collectively, in this review we describe the Universe of EVPs, while providing evidence that neuroregulatory EVPs convey neuroprotective and neurodegenerative effects. The diversity of EVP populations reflects their origin, complexity, and roles in multiple neurological processes. While an unprecedented amount of scientific work has propelled the development of cell-based strategies to protect and repair the injured brain. Current clinical outcomes from treatments in patients’ psychiatric and neurodegenerative disorders such as Alzheimer’s, Parkinson’s disease or schizophrenia, have not reached the anticipated and strong success of preclinical trials. Patient heterogeneity and lack of standardized procedures might have contributed to this adverse scenario. Immune response, immune compatibility, dosing, and administration route may help explain the limited translatability of current therapies. EVPs have brought excitement to new diagnostics and therapeutical alternatives due the incremental evidence of their role in signal transmission and amplification as vehicles of cell communication, healthy phenotypes, aging and pathological states and diseases.

Therefore, there is a critical need to further characterize the function of the neurosecretome with the aim to characterize mechanisms that allow to sustain recovery in psychiatric and neurodegenerative disorders. In the clinical and preclinical settings, it is essential to identify the vesicles, particles and their subpopulations carrying novel and key molecules or group of molecules responsible for the known therapeutic effects, define the relationship between cells and their secreted factors, and elucidate the temporal window of application and dynamics of release.

A major argument in the field is whether there are biomarkers associated with brain derived EVPs and if so then how is one to evaluate their presence in the biofluids. In this context, we evaluated over ∼20 different studies that have isolated brain-derived EVPs from various biofluids (blood, serum, plasma, CSF, tissue, and *in-vitro* tissue cultures) using different EVP isolation methods. A detailed list of brain-derived biomarkers that are being studied in EVPs is provided on Table 1. This comprehensive and cumulative study allowed us to envision brain derived EVPs with a global view. Primarily, Aβ_42_ (Li et al., 2022), P-tau (Palmqvist et al., 2020), APOE4 (for AD) (Palmqvist et al., 2023), α-synuclein (for PD) (Niu et al., 2020), many miRNAs (for AD (Wiedrick et al., 2019), addiction (Chand et al., 2021), SCZ (Du et al., 2019), GBM (Ebrahimkhani et al., 2018)) were discovered in the isolated EVPs. Although most published studies on Table 1 suggested the presence of brain-derived biomarkers in the systemic circulation, it was not entirely clear if there is a linear correlation between the biomarkers and the disease. However, given the diversity of the isolation methods and biofluids it is difficult to come to concrete conclusion. Overall, emerging evidence suggest that brain derived biomarkers are present in the various EVPs and we’re embarking on the journey of reproducible detection and direct correlation of EVPs with brain cancers, psychiatric, neurodegenerative diseases. The incremental knowledge derived from neurosecretome studies will help to design improved liquid-biopsy assays and therapies either using EVPs or their bioengineering analogs to deliver efficiently beneficial molecules for repairing the injured brain. Innovative and groundbreaking research has unveiled the immense potential of EVPs in treating psychopathological phenotypes, paving the way for an exciting new era of clinical benefits for patients.
